# Bird Communities of the Arctic Shrub Tundra of Yamal: Habitat Specialists and Generalists

**DOI:** 10.1371/journal.pone.0050335

**Published:** 2012-12-11

**Authors:** Vasiliy Sokolov, Dorothée Ehrich, Nigel G. Yoccoz, Alexander Sokolov, Nicolas Lecomte

**Affiliations:** 1 Institute of Plant & Animal Ecology, Ural Division Russian Academy of Sciences, Ekaterinburg, Russia; 2 Department of Arctic and Marine Biology, University of Tromsø, Tromsø, Norway; 3 Ecological Research Station of the Institute of Plant & Animal Ecology, Ural Division Russian Academy of Sciences, Labytnangi, Russia; Universidad Nacional Autonoma de Mexico, Mexico

## Abstract

**Background:**

The ratio of habitat generalists to specialists in birds has been suggested as a good indicator of ecosystem changes due to e.g. climate change and other anthropogenic perturbations. Most studies focusing on this functional component of biodiversity originate, however, from temperate regions. The Eurasian Arctic tundra is currently experiencing an unprecedented combination of climate change, change in grazing pressure by domestic reindeer and growing human activity.

**Methodology/Principal Findings:**

Here we monitored bird communities in a tundra landscape harbouring shrub and open habitats in order to analyse bird habitat relationships and quantify habitat specialization. We used ordination methods to analyse habitat associations and estimated the proportions of specialists in each of the main habitats. Correspondence Analysis identified three main bird communities, inhabiting upland, lowland and dense willow shrubs. We documented a stable structure of communities despite large multiannual variations of bird density (from 90 to 175 pairs/km^2^). Willow shrub thickets were a hotspot for bird density, but not for species richness. The thickets hosted many specialized species whose main distribution area was south of the tundra.

**Conclusion/Significance:**

If current arctic changes result in a shrubification of the landscape as many studies suggested, we would expect an increase in the overall bird abundance together with an increase of local specialists, since they are associated with willow thickets. The majority of these species have a southern origin and their increase in abundance would represent a strengthening of the boreal component in the southern tundra, perhaps at the expense of species typical of the subarctic zone, which appear to be generalists within this zone.

## Introduction

Bird communities and populations are monitored in many parts of the world, as they often are the focus of considerable interest from the general public. Their abundance and distributions are considered effective indicators of changes in biodiversity, habitat quality and availability [1]. Bird communities are indeed strongly related to habitat characteristics [2], and this habitat specificity is an important component in explaining and predicting the response of bird communities to environmental changes [3], [4], [5]. In particular, habitat specialists seem to be more negatively affected by environmental changes as they are declining in many areas of the world, whereas generalists are often increasing [1], [6], [7], [8]. A likely consequence is a gradual homogenization of biodiversity [9], [10], [11], a phenomenon that may not be apparent if one focuses on e.g. species richness as an index of diversity [12]. Including functional components of diversity such as degree of specialization has therefore been stressed in recent studies of impacts of global changes [13].

Most studies investigating the response of bird communities to environmental changes have been carried out in temperate regions, and little is known on how the identified trends – homogenization of biodiversity [13] and decline of specialists in favour of increased abundance of generalists [1], [7], [8] – can be translated to Arctic ecosystems [14], [15]. However, in the Arctic, a region usually considered as relatively pristine, the environment is now changing through a combination of climate change and increased human activity [16], [17]. Shrubs are expanding in the southern tundra as a consequence of climate warming [18], [19]. Intense grazing by reindeer/caribou (*Rangifer tarandus*) can, however, limit shrub expansion [20], and may even lead to a decrease of willow shrub cover when densities are particularly high [21], [22], [23]. Grazing induced loss of shrubs has been shown to strongly reduce bird species richness in northern Norway [24]. In addition, increased human activity in tundra areas related notably to oil/gas exploitation leads to increased disturbance, habitat fragmentation and erosion of some key tundra habitats [25]. How these habitat changes affect the different components of tundra ecosystems is however still unclear [23]. Understanding bird habitat associations will improve our understanding of the likely impacts of different components of global change on these communities, but these associations and their spatio-temporal variation are as far as we know very poorly known, with only a handful of studies done in the low Arctic [24], [26], [27].

In this paper, we investigate habitat associations and the degree of habitat specialization of bird communities in the shrub tundra of the south-western Yamal peninsula, Russia. This region is experiencing both a rapid development of the oil and gas industry, and growth of reindeer herds [28]. Climate change and associated geomorphologic processes such as permafrost melting have an increasing impact on ecosystem processes [16], [29], [30]. Bird communities on Yamal peninsula and species habitat preferences had been described already by Zhitkov [31] and Sdobnikov [32]. Uspenskyi [33] highlighted some biogeographic aspects and Danilov et al. [34] characterized the bird communities typical for different latitudinal zones, together with their associated landscape elements. These works were mostly faunistic, however, and no quantitative, multi-annual study of habitat associations and their stability over time, i.e. specialization, exists.

Here we present results of a systematic bird survey carried out over eight years (2002–2009) in five habitat types of the shrub tundra in southern Yamal, habitats that are typical for vast areas in the southern Eurasian Arctic [30]. We first use multivariate statistics to analyse associations of bird species to habitats and identify habitat specific communities. Second, we quantify the specialization of bird communities in the main habitats. In particular, we address the importance of willow (*Salix* spp.) thickets for the bird communities, as their extent in the shrub tundra is likely to change under the influence of climate change, erosion and/or intense browsing. Willow thickets have been described as hotspots of productivity and biodiversity in general [23], [24], [35], [36], with a positive effect on bird species richness in particular [24], [35]. Higher bird densities and higher species richness would thus be expected in this habitat. As willow thickets are a habitat component with characteristics from more southern climatic zones such as the forest tundra, one could in addition expect that the thickets harbor a higher number of species whose main distribution area is south of the tundra. Here we focus on the most common species in the area, mostly songbirds.

## Materials and Methods

### Ethics statement

The study was conducted in the frame of ecosystem monitoring carried out by the Ecological Research Station of the Institute of Plant & Animal Ecology, Ural Division Russian Academy of Sciences, and part of the approved science plan of this institution. Permissions for field work were obtained from the Department of Bioresource of the Government of Yamalo-Nenetsky Autonomous Okrug (the administrative region where the study was carried out). As this was a purely observational study, no specific permits were needed.

### Study area and habitat classification

**Figure 1:**
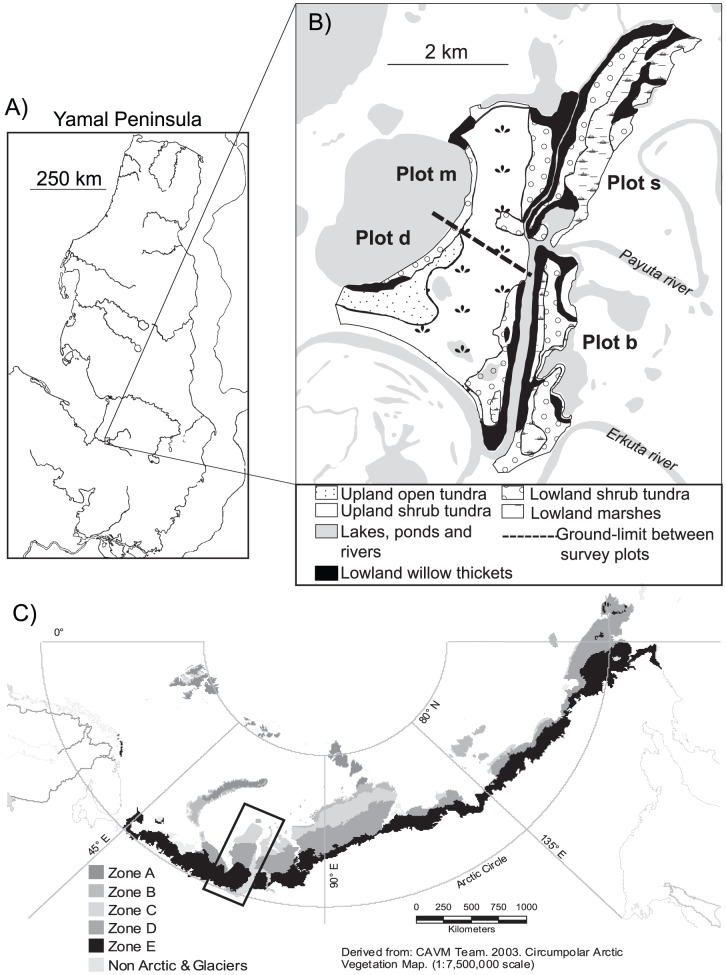
Maps showing the study area in Yamal, Russia. A) the study area divided into four study plots with the extent of the five different habitat types, B) the location of Erkuta tundra monitoring site in southern Yamal and C) the location of Yamal in the Eurasian Arctic with the five arctic bioclimatic subzones as used by Walker *et al.* (2005).

Data were collected during a long-term study of birds at the Erkuta tundra monitoring site, situated close to Erkutayakha River in southwest Yamal (68°13′N 69°09′E), Russia (Figure 1). Mean temperature in this area is −25.7°C in January and 8.6°C in July [37] (World Meteorological Organisation). Daily average temperatures become positive in the first decade of June and negative again around the first week of October. On average, precipitation is about 350 mm per year and falls mainly as rain in summer. A stable snow cover is usually established in early October and lasts until early June.

The study area is situated in a flat tundra landscape interspersed with hills (ca. 30 m) and river cliffs (up to 40 m high). A dense network of rivers, streams and lakes creates wide lowlands with large areas being flooded in spring. The area is at the border between two vegetation zones: erect dwarf-shrub and low-shrub tundra [30]. Low shrub tundra is more common in the area than the drier, lichen-rich erect dwarf-shrub tundra [38]. Plant cover is typically continuous (80–100%), but may be sparse (5–50%) on dry ridges. Dense thickets composed of willows and in some places alder (*Alnus fruticosa*) occur along streams and lakes.

The bird survey was carried out in an area covering 3.2 km^2^. After an initial survey of a larger area of about 100 km^2^ the study area was chosen because 1) it contained all major landscape elements typical for the region, elements which are also characteristic of the southern tundra in Russia in general (Walker et al. 2005), and 2) the size of the area was small enough to carry out the survey several times per season and by the same observer. The area was divided into four plots of about 0.8 km^2^ each, delimited mostly by landscape elements such as rivers or lakes, to assess the local variation in bird communities (Figure 1). The plots were divided into five habitats according to the landscape elements and vegetation types (Table 1). The main landscape elements in the area include “uplands” which consist of flat tundra on hills and their slopes and “lowlands” which are usually flooded in spring. Based on vegetation types as mapped by S. N. Ektova in 2004 [38], the distribution of bushes (*Salix spp*, *Betula nana*) and smaller shrubs (*Empetrum nigrum, Ledum palustris*), as well as moisture, we distinguished two upland habitats: upland open tundra (UOT) and upland shrub tundra (UST; See Figure S1 for pictures of the habitats). Lowland habitats were divided into lowland shrub tundra (LST) and lowland marshes (LM). As shrubs in general and willow thickets in particular are important structural elements and highly productive patches in the tundra ecosystem [22], [39], [40], [41], the dense willow thickets (up to 2.5 m) growing along rivers and in flooded areas were classified as a distinct habitat type occurring on lowlands (WT). These five habitats comprise different structural elements determining breeding habitat and differ in resource availability. Several habitat types were found on each of the four plots, but usually not all five. In total, the area comprised 14 habitat x plot units (Figure 1).

**Table 1:** Characteristics of the five habitat types used to determine bird-habitat associations in southern Yamal, Russia.

Habitat type	Landscape/Microrelief	Dominant vegetation	Flooded
Upland open tundra	Tussock tundra	Sedge (*Carex arctosibirica*.)	No
(UOT)		cottongrass (Eriophorum spp.)	
Upland shrub tundra	High/low centred polygons	Shrubs (*Betula nana, Salix glauca & phylicifolia*)	No
(UST)	scattered patterned bare ground	dwarf shrubs (Vaccinium spp., *Empetrum nigrum*)	
	tussocks/hummocks	sedge, grass (*Calamagrostis lapponica*)	
Low shrub tundra	Slopes/lowlands,polygons	Shrubs, dwarf shrubs, cloudberry (*Rubus chamaemorus*))	Yes
(LST)	scattered tussocks/hummocks	sedge, grass, Labrador tea (*Rhododendron tomentosum*)	
Lowland marshes	Lowland marshes	Sedge *C. aquatilis*, moss (*Sphagnum sp.*), shrubs	Yes
(LM)		dwarf shrubs, grass	
Willow thickets	River/lakes bank	Thicket forming willows (*Salix lannata*, S. pulchra)	Yes
(WT)	lowland marshes	sedges, grass moss	

### Bird surveys

Birds were surveyed during the breeding season from middle of June to middle of July in 2002–2009 using the spot mapping method [42], [43], [44]. An 8- year of survey covered the large year-to-year variation in phenology, weather and small rodent abundance. Each plot was surveyed by walking back and forth at a slow pace along tracks 100 m apart, recording all alarming or singing birds, at least four times in each breeding season by the same observer (V. A. Sokolov). A distance of 100 m between tracks was chosen because up to 50 m distance it is possible to observe and identify birds with good confidence in open habitat. At the same time, given the average territory sizes of birds in the region (Ryabitsev 1993) and typical densities (Methods S1; Figure S2), this distance minimizes the chances for double counting. Tracks were also always placed along thicket edges, allowing for good coverage of this habitat with less visibility and higher densities (see Methods S1 for more details). Limits between plots were located with a handheld Global Positioning System unit (GPS; Garmin eTrex, accuracy 5 meters). The location of each bird was recorded by GPS and each observation was subsequently plotted on a topographic map.

All male/female pairs were noted and recorded as breeding pairs. An alarming or singing male was assumed to represent a breeding pair within the plot, but was recorded as a pair only if it was observed more than once at the same place during the season. For some species, we used additional methods to determine the number of breeding pairs: nest searching for abundant species (Red-throated Pipit, Lapland Bunting; Latin names for all species are given in Table 2) and point counts for some shrub species (e.g. Willow Warbler, Redwing, see also Methods S1). All plots were surveyed in approximately the same weather conditions and mostly in the early morning (from 4 to 9 AM) and evening (from 5 to 8 PM), thus at times when the activity of the birds is likely to be high. Surveys were not conducted during periods of rain, strong winds, or restricted visibility (e.g. fog). Densities of each species were calculated as the number of breeding pairs per km^2^.

**Table 2:** Mean annual densities (pairs/km^2^ with standard errors based on variation among years) of birds observed at the Erkuta monitoring site in 2002–2009 by habitat type.

	Code			Habitat type			Overall	SSI	Distr	Nest
		UOT	UST	LST	LM	WT				
Willow Grouse	Lag.lag	0	2.0 (0.6)	6.5 (1.5)	4.7 (1.0)	3.0 (1.3)	3.2 (0.5)	0.52	SA	T, O
*Lagopus lagopus*										
Golden Plover	Pl.apr	0	1.1 (0.2)	0.3 (0.2)	0	0	0.3 (0.1)	1	SA	O
*Pluvialis apricaria*										
Ringed Plover	Ch.hyat	0.5 (0.5)	0.1 (0.1)	0.1 (0.1)	0	0	0.2 (0.1)	0	SA	O
*Charadrius hiaticula*										
Wood Sandpiper	T.glar	1.5 (0.7)	1.9 (0.3)	6.7 (0.8)	5.6 (0.9)	5.2 (1.3)	4.2 (0.3)	0.33	S	T, O
*Tringa glareola*										
Red-Necked Phalarope	Ph.lob	0	0.3 (0.2)	4.1 (0.7)	3.2(1.3)	3.7 (1.1)	2.2 (0.5)	0.56	SA	O
*Phalaropus lobatus*										
Ruff	Ph.pug	0	0.4 (0.2)	2.6 (0.5)	4.7(1.6)	2.8 (0.6)	2.1 (0.4)	0.58	W	T, O
*Philomachus pugnax*										
Temminck's Stint	C.tem	4.0 (1.5)	1.9 (0.5)	8.6 (1.1)	3.4(1.1)	7.8 (2.0)	5.1 (0.6)	0.35	SA	O
*Calidris temmincki*										
Jack Snipe	L.min	0	0	0.7 (0.3)	2.5(0.7)	0.9 (0.5)	0.8 (0.2)	0.69	S	T, O
*Lymnocryptes minimus*										
Common Snipe	G.gal	0	0.1 (0.1)	5.1 (1.1)	9.3(2.5)	4.3 (0.7)	3.8 (0.7)	0.79	S	T, O
*Gallinago gallinago*										
Arctic Tern	St.parad	0	0.4 (0.2)	0	0	0	0.1 (0.03)	1.02	SA	O
*Sterna paradisaea*										
Shore Lark	E.alp	2.0 (0.8)	1.2 (0.5)	0.4 (0.3)	0	0	0.7 (0.2)	0.63	SA	O
*Eremophila alpestris*										
Pechora Pipit	A.gust	0	0	1.9 (0.8)	1.7 (0.4)	2.6 (0.6)	1.2 (0.3)	0.53	S	T
*Anthus gustavi*										
Meadow Pipit	A.prat	8.5 (1.2)	4.7 (0.8)	1.5 (0.4)	1.0 (0.6)	0.2 (0.2)	3.2 (0.3)	0.81	S	T, O
*Anthus pratensis*										
Red-Throated Pipit	A.cerv	18.0 (3.4)	14.1 (2.6)	26.2 (3.8)	17.4 (2.6)	15.7 (3.8)	18.3 (2.6)	0.14	SA	T, O
*Anthus cervinus*										
Citrine Wagtail	M.citr	0	0.3 (0.2)	5.7 (1.0)	4.7 (1.2)	8.2 (1.8)	3.8 (0.6)	0.7	S	T
*Motacilla citreola*										
Yellow Wagtail	M.flava	0	0.1 (0.1)	1.9 (0.4)	2.5 (0.5)	1.7 (0.7)	1.2 (0.2)	0.48	S	T, O
*Motacilla flava*										
Pied Wagtail	M.alba	0.5 (0.5)	1.7 (0.4)	0.4 (0.2)	0	1.3 (0.6)	1.2 (0.2)	0.42	W	T, O
*Motacilla alba*										
Sedge Warbler	A.schoen	0	0	0.9 (0.2)	2.5 (0.9)	7.5 (1.3)	2.0 (0.4)	1.12	S	T
*Acrocephalus schoenobaenus*										
Willow Warbler	Ph.troch	0	0.7 (0.3)	5.1 (0.9)	3.2 (0.6)	29.7 (4.2)	7.8 (0.9)	1.43	S	T
*Phylloscopus trochilus*										
Chiffchaff	Ph.coll	0.5 (0.5)	0.4 (0.2)	2.2 (0.7)	1.2 (0.5)	10.8 (3.1)	3.0 (0.5)	1.17	S	T
*Phylloscopus collybita*										
Arctic Warbler	Ph.bor	0	0	0	0	0.4 (0.4)	0.1 (0.1)	0.57	S	T
*Phylloscopus borealis*										
Northern Wheatear	O.oen	3.0 (1.3)	0.6 (0.3)	0	0	0	0.7 (0.2)	1.09	W	T
*Oenanthe oenanthe*										
Bluethroat	L.svec	0	2.2 (0.4)	7.4 (1.2)	1.7 (0.6)	10.6 (1.4)	4.4 (0.4)	0.79	S	T
*Luscinia svecica*										
Redwing	T.iliacus	0	0.4 (0.2)	2.0 (0.4)	0.7 (0.4)	13.3 (1.5)	3.4 (0.4)	1.44	S	T
*Turdus iliacus*										
Redpoll	A.flam	0	0.6(0.2)	8.4 (0.5)	3.9 (1.3)	24.4 (3.2)	7.5 (0.7)	1.17	SA	T
*Acanthis flammea*										
Reed Bunting	E.schoen	0	0.2 (0.2)	1.9 (0.6)	2.9 (0.7)	4.7 (1.4)	2.0 (0.3)	0.66	S	T
*Emberiza schoeniclus*										
Little Bunting	E.pus	0.5 (0.5)	2.5 (0.9)	9.4 (2.0)	7.1 (1.3)	27.2 (3.9)	9.3 (1.0)	0.97	S	T, O
*Emberiza pusilla*										
Lapland Bunting	C.lap	6.0 (1.3)	16.8 (1.4)	12.6 (3.0)	10.8 (1.9)	4.1 (0.8)	10.1 (1.0)	0.38	SA	O
*Calcarius lapponicus*										

The overall density in the study area is given, as well as the species specialization index (SSI, corrected for sample size, see main text for details). Distr refers to the distribution type of the species (SA = subarctic distribution; S = southern; W = widespread) and Nest to the general nesting habitat (T = thicket; O = open) as reported by [34].

### Data analyses

Counts of the most common and noticeable species (28 species, Table 2) on the four study plots were analysed in order to characterize bird-habitat relationships. Community composition was examined using Correspondence Analysis (CA) and its extensions [45], [46]. These ordination analyses allow for a comparison of the relative abundance of species within a community [47] and we therefore did not correct for the surface covered by the different habitats. Analysing the whole data set, we assessed how much of the overall variation was due to differences among habitats (5 habitats), plots (4 plots) and years (8 years), using Canonical Correspondence Analysis (CCA) [46], [48]. The percentage of variation explained is based on comparing eigenvalues obtained from the unconstrained ordination (CA) and the constrained CCA.

To determine the proportion of specialists in each habitat, an index of habitat specialization (SSI) was calculated for each species following Julliard et al. [49]. Specialization was quantified as the coefficient of variation of the average densities of a species in each of the five habitats. As sample size was small for some species, we assessed the bias correction suggested by Devictor et al. [8]. This correction was based on two approximations: a Poisson distribution within each habitat class and assuming identical habitat frequencies. However, the distribution within habitat classes is in fact multinomial when conditioning on total number of observed birds, and unequal habitat frequencies as in our study will increase the variance among habitat classes. We therefore calculated the bias by simulating samples from a multinomial distribution with frequencies based on our study area, and deriving the expected SSI for a perfect generalist (Devictor et al 2008b). The observed SSI values were then corrected by the estimated bias.

A community specialization index (CSI) was estimated for the birds in each habitat on each plot (14 habitat x plot units). CSI was calculated as the average SSI of the individuals counted in that habitat/plot over the years of the survey [49]. The species were further classified according to their distribution type as subarctic, southern, or widespread (Table 2; Danilov 1966). Subarctic species are species, which have evolved in the subarctic, whereas southern species are mainly distributed south of the tundra, but extend into the southern part of the Arctic [50]. Widespread species have a distribution encompassing several bioclimatic zones (e.g. ruff, wheatear or pintail) [48]. For each habitat/plot, we calculated the proportion of individual birds belonging to each distribution type among the birds counted in that habitat/plot to assess whether birds with a particular distribution favoured specific habitats.

Average species richness for each habitat was estimated applying the first order jackknife estimator to the bird counts on each habitat in each year, and using plots as replicates [51]. All statistical analyses were conducted using the open-source software R, version 2.11 [52] and the libraries ade4 for multivariate analysis [53] and vegan for species richness estimation [54].

## Results

### Abundance

The overall density of breeding birds in the study area fluctuated from 90 to 175 pairs per km^2^ over the years (average 108.7±10.4 (SE) pairs/km^2^). In total, 41 species were recorded as breeders. Several species occurred however at very low densities or were recorded only once. These were the following (alphabetical order): Rough-legged Buzzard *Buteo lagopus*, Black-throated Diver *Gavia arctica*, Red-throated Diver *Gavia stellata*, Long-tailed Duck *Clangula hyemalis*, Bean Goose *Anser fabalis*, Greater White-fronted Goose *Anser albifrons*, Heuglin's Gull *Larus heuglini*, Pintail *Anas acuta*, Greater Scaup *Aythya marila*, Common Scoter *Melanitta nigra*, Arctic Skua *Stercorarius parasiticus*, Common Teal *Anas crecca*, and Wigeon *Anas Penelope*.

In the following, we analysed the 28 most common species (Table 2 with Latin name of the species mentioned below). The Red-throated Pipit was the most abundant species on the study plots, with an average of 19.4 pairs/km^2^ (Table 2). It was almost twice as abundant as the next most common species, the Lapland Bunting (12.5 pairs/km^2^ on average). Little Bunting, Redpoll, Willow Warbler, Bluethroat, Redwing, Citrine Wagtail, Meadow Pipit and Chiffchaff were common, but less abundant. Among waders, the most common species were Temminck's Stint, Wood Sandpiper as well as Common Snipe, with densities ranging from 4.5 to 5.4 pairs/km^2^ (Table 2). Willow Grouse was also rather common. Other birds had densities less than 3 pairs/km^2^, and several species, such as Golden Plovers, Ringed Plovers or Shore Larks were not recorded every year.

### Variation in bird community compositions

**Figure 2:**
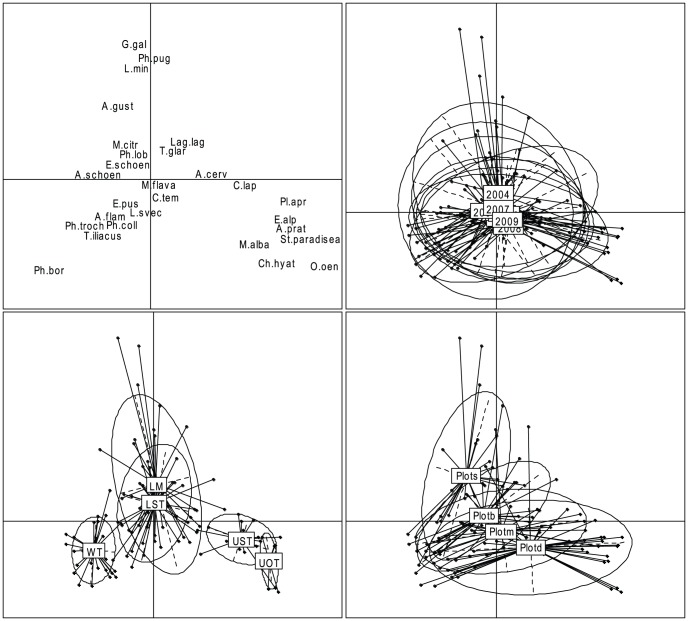
Correspondence analysis of bird communities of southern Yamal, Russia. Species scores (upper left), variation between years (upper right), habitats (bottom left) and plots (bottom right). Ellipses describe the variability within habitats and plots and have an approximate 67% confidence level. Habitat and species codes are given in Tables 1 and 2, respectively.

The two first axes of the CA clearly represented much larger components of variation than the following axes (Axis 1 and 2: 23% and 13% of variation respectively, all other axes <6%). These two first axes reflected the difference in bird composition among three types of habitats, the upland tundra (UOT and UST), the lowland flooded tundra (LST and LM), and willow thickets (WT; Figure 2). Despite considerable fluctuations of overall population density from year to year, fluctuations of community composition among the years were small compared to variation among habitats and plots. Compared to the two first unconstrained eigenvalues of CA (0.36 and 0.22), the two first eigenvalues of a constrained CA with year as categorical covariate were both 0.02, with habitat as a covariate 0.31 and 0.11, and with plot as a covariate 0.17 and 0.04.

**Figure 3:**
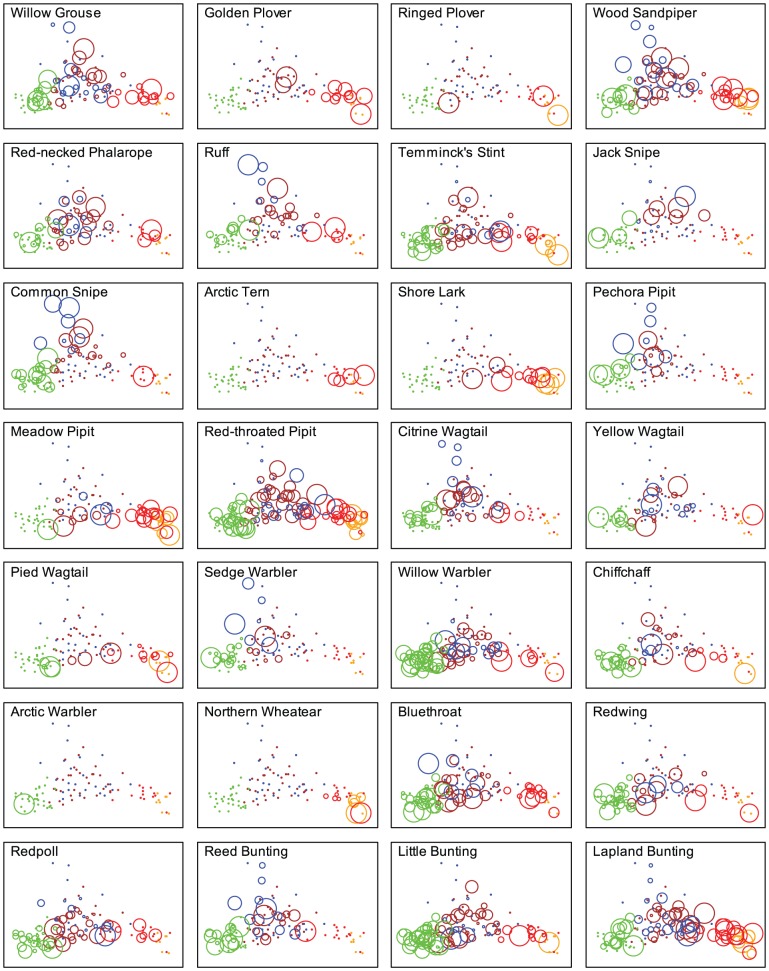
Relative abundance of each species across habitats. Correspondence analysis is used to define the coordinates of each plot-habitat-year observation, and different colours indicate different habitats (orange – upland open tundra, red – upland shrub tundra, brown – lowland shrub tundra, blue – lowland marshes and green – willow thickets). The size of the circles is proportional to the abundance of the given species in the respective plot-habitat-year.

We also investigated whether an interaction between year and habitat could explain some additional variation, but this was not the case (first two eigenvalues of CA with the interaction habitat*year as a covariate: 0.33, 0.13, compared to 0.31 and 0.11 with habitat only). The species composition overlapped largely between the four plots and differences were largely confounded with the different habitats present on each plot. Generalist species, characterized by a large variance along the two first CA axes, were Wood Sandpiper, Temminck's Stint, Red-throated Pipit, and Lapland Bunting (Figure 3). These species were found in nearly all habitat types almost every year. Species specializing on upland tundra as their main habitat were Golden Plover, Ringed Plover, Arctic Tern, Shore Lark, Meadow Pipit and Northern Wheatear. The flooded lowland areas (LST and LM) were preferred by Willow Grouse, Red-necked Phalarope, Ruff, Jack Snipe, Common Snipe, Pechora Pipit, Yellow Wagtail and Citrine Wagtail. Sedge Warbler, Willow Warbler, Chiffchaff, Arctic Warbler, Bluethroat, Redwing, Redpoll, Reed Bunting and Little Bunting exhibited a clear affinity to willow thickets as habitat (Figure 3).

### Variation in specialization among habitats

**Figure 4:**
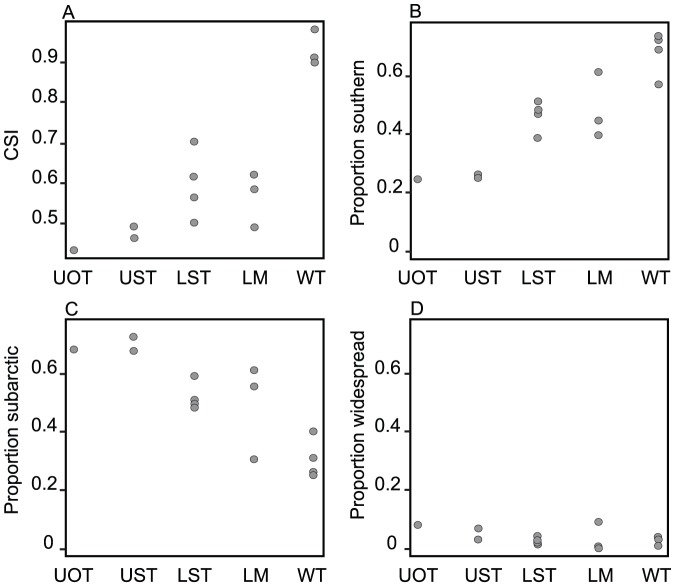
Comparison of the bird communities in five habitat types. UOT – Upland open tundra, UST – upland shrub tundra, LST – lowland shrub tundra, LM – lowland marshes, WT – willow thickets. A) Community specialization index, B) proportion of birds belonging to species with a southern distribution, C) proportion of birds belonging to species with a subarctic distribution and D) proportion of birds belonging to widespread species.

The gradient from generalists to specialists was well described by SSI (Table 2). The Red-throated Pipit, the most common and widespread species in the area, had the lowest value (0.25). The Temminck's Stint and Wood Sandpiper had low values as well (0.57 for both). The most specialized species were Willow Warbler, Redwing, Redpoll, Chiffchaff, and Little Bunting (in decreasing order of specialization; Table 2). Birds representing specialized species were on average most numerous in willow thickets, which had the highest CSI. CSI was, in contrast, lowest for the two upland habitats (UOT and UST) and intermediate for the open lowland areas (LST and LM; Figure 4). The proportion of species with a southern distribution showed a similar pattern as CSI (Figure 4). Willow thickets harboured most southern species and had the highest CSI, indicating that species with a southern distribution are habitat specialists in the shrub tundra preferring willow thickets. The proportion of subarctic species was inversely proportional to that of southern species, and most subarctic species occurred in the upland habitats (UOT and UST). Species with a wide distribution range represented only a small proportion of the species in all habitats (Figure 4).

Among the five habitats, the density of breeding birds was clearly highest in willow thickets (Table 3). Estimates of species richness did not change much over the years; therefore the values were averaged across years to get an overall species richness estimate for each habitat. Species richness was not higher in willow thickets than in the two other lowland habitats LST and LM (Table 3). Both density and species richness were lowest in UOT.

**Table 3:** Bird densities and species diversity in five different habitats of the shrub tundra of southern Yamal, Russia.

Tundra habitat	Upland open	Upland shrub	Lowland shrub	Lowland marshes	Willow thickets
Area (km^2^)	0.3	1.2	0.9	0.5	0.6
Nb species	11	24	25	21	23
Total density	37.5 (9.0)	54.4 (18.4)	117.2 (34.8)	96.5 (23.5)	183.8 (57.5)
Species richness	5.4 (1.5)	17.8 (3.7)	25.6 (3.6)	22.3 (3.5)	23.1 (3.5)

Subscripts: Total area of the habitat on the four study plots (Area), total number of species observed over the eight study years (Nb species), average density of birds in the habitat when summing all species (Total density, standard deviations reflecting variation among years are given in brackets), and species richness (standard deviations reflecting variation among years are given in brackets).

## Discussion

As far as we know, relatively few studies have been published on bird communities in the shrub tundra zone, and this is particularly true for passerines [24], [26], [27]. Moreover, because of logistical constraints, previous studies represented often snapshots of one or two years, e.g. [44]. Little is therefore known about the temporal variability of abundance in these bird communities, even though there are indications that it can be high [26], [27]. By surveying the same plots over 8 years (2002–2009), characterized by large fluctuations in abiotic (e.g. snowmelt) and biotic conditions (e.g., small rodent densities) typical for the Arctic (International Waders Study Group 2008), we could analyse fluctuations in composition, abundance, and species richness. We acknowledge that our estimates of population densities can be affected by e.g. variation in detectability and double counts, but these issues are unlikely to impact our conclusions regarding species diversity and community specialization as we used robust estimators.

Compared to studies from the North American Arctic, the number of species registered in the present study was high, particularly so for small passerines. Eighteen small passerine species were breeding at Erkuta, compared to values ranging from eight to nine in a comparable biogeographic zone in eastern Canada [26], [27]. Jackson and Robertson [55] found 14 passerine species in their “oceanic heath/stony ground” zone, which covers the low Arctic zone of northern Norway. The small number of studies prevents any generalization, but one can speculate that low Arctic bird communities in Eurasia are richer than in North America.

Passerine communities at Erkuta differed from North American ones by the occurrence of pipits, wagtails, warblers, as well as one thrush species. Specifically the Red-throated Pipit was an abundant generalist at Erkuta, with two other pipit species being present, whereas the only study we could find from the Canadian Arctic with significant numbers of pipits was on Ungava peninsula where the American Pipit (*Anthus rubescens*) is a willow specialist [26]. In our study, wagtails and warblers were represented by six species, four being rather abundant, whereas Sammler et al. [27] recorded only one species of warbler (of course, new world warblers do not belong to the same taxonomic group as old world warblers, but we consider here the functional role these groups play in the tundra ecosystems). Other components of the community at Erkuta were more similar to passerine assemblages in North America. To the extent they can be compared functionally, buntings (little and reed) could replace the savannah and American tree sparrow that are characteristics of the low Arctic in Canada. Some species are found throughout the circumpolar Arctic such as the Lapland Bunting and Shore Lark. The Lapland Bunting is nearly always a dominant species and occurs in all vegetation types (this study; [17], [26], [27], [56]) except in the high Arctic where Snow Bunting dominates [44]. Horned Lark is also widespread but occurs at low densities throughout the Arctic and is more selective in the choice of habitat (open, often dry tundra). The passerine community at Erkuta was more similar to communities described in northern Norway, notably in the numerical dominance of pipits [14], [24].

Yearly variation in abundance was large, but around values typical of the low Arctic [27]; species composition was, however, stable and mostly determined by differences among habitats. Monitoring of community composition rather than abundance or species richness should therefore give more reliable indications of how environmental changes affect tundra environments. Although the size of our study area was rather small, the number and species of birds present were similar to surveys done within the same bioclimatic zone (e.g. [57], [58], [59]; see also Figure S3). Multivariate analyses distinguished mainly three species assemblages among the five studied habitats. The first assemblage occurred in upland tundra, the drier, open parts of the landscape. This community was characterized by species with a low degree of habitat specialization and by a high proportion of subarctic species. A second, distinct assemblage was found in willow thickets, with the highest specialization index and the most southern species. The third community, which occurred in the flooded lowland tundra (LST and LM), occupied an intermediate place between the upland tundra and the willow thickets on the first axis of the CA, but was distinct on the second axis. It was composed both by southern and subarctic species in about equal proportions, and harboured specialists as well as generalists.

Of all habitats, willow thickets had the highest densities of breeding birds. This high density may be related to the high productivity of willow thickets in terms of plant biomass, possibly resulting in high abundance of arthropods as food for breeding birds. Structurally, the thickets represent sheltered breeding sites both on and above the ground and elevated sites used for display by species like Bluethroats or warblers. Willow thickets had a high value for the specialization index, mainly because southern species in the tundra zone were restricted to willow thickets. However, this influx of southern species did not result in higher species richness as most typical subarctic species were mainly found in other habitats, either lowland or upland tundra.

Community specialization was lowest in the upland habitats, i.e. open and upland shrub tundra, characterized by a relatively simple vegetation structure compared to willow thickets. The dominant species in the upland habitats, such as Lapland Bunting and Red-throated Pipit, were typically species found also in other habitats. Although the community composition of the two upland habitats was similar, it differed more than between the two lowland tundra habitats (LST and LM; Figure 2). Upland open tundra was the habitat with the lowest density of breeding birds as well as the lowest specialization index.

The low Arctic zone in Yamal peninsula is expected to be impacted by three main drivers of change in the next decades: warming, reindeer herding, and oil or gas exploitation [28]. The development of the latter is being most intense further north on Yamal peninsula and is not expected to affect our study area directly [16]. Warming is expected to increase shrub cover [60], and therefore the extent of willow thickets, whereas reindeer grazing will have inverse effects [23], [24], [61]. Reindeer herds on Yamal peninsula have increased considerably during the last 20 years, but it is unknown whether such densities will stay sufficiently high in the future to slow down significantly the expected increase in willow thickets. Assuming a scenario of willow thickets increase, we would expect increasing overall bird abundance, as well as an increase of specialists as defined at the scale of our study, since those species tend to dominate willow thickets. This would be the reverse pattern of what is observed for example in temperate areas, where there is both a specialists decline and a decrease in bird specialization, with specialists becoming generalists [6]. Part of the discrepancy may be due to scaling issues and the variation in habitats considered. Many species considered as specialists in our study area, a classification which is likely to be representative for a large part of the southern Arctic in Russia, would be generalists if boreal habitats were included (e.g. warblers, Little Bunting, Citrine Wagtail or Bluethroat).

This study is a first step to understand bird communities in the large bioclimatic zone of the southern Arctic tundra covering ca 800,000 km^2^ in Russia [30]. The pattern we analyse here warrants studies at a larger scale (for example by including the taiga zone) to understand how our local-scale results may translate to a regional scale, as the degree of specialization may vary across the range of species (e.g. [7]). The main difference with studies done in the temperate zone is due to the fact that we expect major increases in the habitat harbouring the largest number of specialists – this is a different pattern from the changes observed in temperate areas ([1] but see [62] for another example), where habitats with a large number of specialists, specifically traditional farmland and to a lesser degree forests, have been under constant pressure. Furthermore, we do not expect thicket specialists to become more generalist for two reasons: they often have strict nesting requirements (e.g. Redwing) and increase of generalist predators such as Red Fox (*Vulpes vulpes*; [63]) and Hooded Crow (*Corvus cornix*; [64]) is likely to prevent expansion to more open habitats. The suggested pattern of increase in local specialists is likely to concern other also groups of organisms in the Arctic, notably plants, where rare species are often limited to particular habitats and microclimates (hotspots), which may become more common with climate change (Elvebakk 2005).

## Supporting Information

Methods S1 **Details about the survey method.**
(DOC)

Figure S1 **Summer pictures of the habitats monitored in our study, Erkuta, 2002–2009, Yamal, Russia.**
(TIF)

Figure S2 **Map of breeding pairs for two species breeding in open habitat (Lapland bunting) and closed habitats (willow thickets; little bunting).** Each star represents the centre of a territory.
(TIF)

Figure S3 **Bird communities have been described at several sites on the Yamal Peninsula by different authors.** A) Map of the sites where communities were described. B) Result of a correspondence analysis which shows that the community at Erkuta was similar to those observed at sites located in the same biogeographic area, such as Hanovey and Yuribey (Sokolov et al. 2006).
(TIF)

## References

[1] Gregory RD, van Strien A (2010) Wild bird indicators: using composite population trends of birds as measures of environmental health. Ornithological Science 9: 3–22.

[2] Wiens J (1989) The ecology of bird communities. 1 Foundations and patterns. Cambridge: Cambridge University Press. 539 p.

[3] Hausner VH, Yoccoz NG, Ims RA (2003) Selecting indicator traits for monitoring land use impacts: Birds in northern coastal birch forests. Ecological Applications 13: 999–1012.

[4] Niemi GJ, McDonald ME (2004) Application of ecological indicators. Annual Review of Ecology Evolution and Systematics 35: 89–111.

[5] Reif J, Storch D, Vorisek P, Stastny K, Bejcek V (2008) Bird-habitat associations predict population trends in central European forest and farmland birds. Biodiversity and Conservation 17: 3307–3319.

[6] Barnagaud JY, Devictor V, Jiguet F, Archaux F (2011) When species become generalists: on-going large-scale changes in bird habitat specialization. Global Ecology and Biogeography 20: 630–640.

[7] Devictor V, Julliard R, Clavel J, Jiguet F, Lee A, et al (2008) Functional biotic homogenization of bird communities in disturbed landscapes. Global Ecology and Biogeography 17: 252–261.

[8] Devictor V, Julliard R, Jiguet F (2008) Distribution of specialist and generalist species along spatial gradients of habitat disturbance and fragmentation. Oikos 117: 507–514.

[9] Clavel J, Julliard R, Devictor V (2011) Worldwide decline of specialist species: toward a global functional homogenization? Frontiers in Ecology and the Environment 9: 222–228.

[10] Davey CM, Chamberlain DE, Newson SE, Noble DG, Johnston A (2012) Rise of the generalists: evidence for climate driven homogenization in avian communities. Global Ecology and Biogeography 21: 568–578.

[11] Olden JD (2006) Biotic homogenization: a new research agenda for conservation biogeography. Journal of Biogeography 33: 2027–2039.

[12] Filippi-Codaccioni O, Devictor V, Bas Y, Julliard R (2010) Toward more concern for specialisation and less for species diversity in conserving farmland biodiversity. Biological Conservation 143: 1493–1500.

[13] Clavel J, Julliard R, Devictor V (2010) Worldwide decline of specialist species: toward a global functional homogenization? Frontiers in Ecology and the Environment

[14] Järvinen O, Väisänen R (1979) Changes in bird populations as criteria of environmental changes. Holoarctic Ecology 2: 75–80.

[15] Virkkalaa R, Heikkinen RK, Leikola N, Luoto M (2008) Projected large-scale range reductions of northern-boreal land bird species due to climate change. Biological Conservation 1343–1353.

[16] Forbes BC, Stammler F, Kumpula T, Meschtyb N, Pajunen A, et al (2009) High resilience in the Yamal-Nenets social-ecological system, West Siberian Arctic, Russia. Proceedings of the National Academy of Sciences 106: 22041–22048. 10.1073/pnas.0908286106 PMC2791666 20007776

[17] Liebezeit JR, Kendall SJ, Brown S, Johnson CB, Martin P, et al (2009) Influence of human development and predators on nest survival of tundra birds, Arctic Coastal Plain, Alaska. Ecological Applications 19: 1628–1644. 19769108 10.1890/08-1661.1

[18] Sturm M, Racine C, Tape K (2001) Climate change - Increasing shrub abundance in the Arctic. Nature 411: 546–547. 11385559 10.1038/35079180

[19] Tape K, Sturm M, Racine C (2006) The evidence for shrub expansion in Northern Alaska and the Pan-Arctic. Global Change Biology 12: 686–702.

[20] Post E, Pedersen C (2008) Opposing plant community responses to warming with and without herbivores. Proceedings of the National Academy of Sciences 105: 12353–12358. 10.1073/pnas.0802421105 PMC2527915 18719116

[21] Bråthen KA, Ims RA, Yoccoz NG, Fauchald P, Tveraa T, et al (2007) Induced shift in ecosystem productivity? Extensive scale effects of abundant large herbivores. Ecosystems 10: 773–789.

[22] den Herder M, Virtanen R, Roininen H (2008) Reindeer herbivory reduces willow growth and grouse forage in a forest-tundra ecotone. Basic and Applied Ecology 9: 324–331.

[23] Ims RA, Yoccoz NG, Bråthen KA, Fauchald P, Tveraa T, et al (2007) Can reindeer overabundance cause a trophic cascade? Ecosystems 10: 607–622.

[24] Ims RA, Henden JA (2012) Collapse of an arctic bird community resulting from ungulate-induced loss of erect shrubs. Biological conservation 149: 2–5.

[25] Forbes BC, Fresco N, Shvidenko A, Danell K, Chapin FS (2004) Geographic variations in anthropogenic drivers that influence the vulnerability and resilience of social-ecological systems. Ambio 33: 377–382. 15387078 10.1579/0044-7447-33.6.377

[26] Andres BA (2006) An Arctic-breeding bird survey on the northwestern Ungava Peninsula, Québec, Canada. Arctic 59: 311–318.

[27] Sammler JE, Andersen DE, Skagen SK (2008) Population trends of tundra-nesting birds at Cape Churchill, Manitoba, in relation to increasing goose populations. Condor 110: 325–334.

[28] Walker DA, Leibman MO, Epstein HE, Forbes BC, Bhatt US, et al (2009) Spatial and temporal patterns of greenness on the Yamal Peninsula, Russia: interactions of ecological and social factors affecting the Arctic normalized difference vegetation index. Environmental Research Letters 4: 045004.

[29] Golovatin M, Morozova L, Ektova S, Paskalny S (2010) The change of tundra biota at Yamal peninsula (the North of the Western Siberia, Russia) in connection with anthropogenic and climate shifts. In: Gutierrez B, Pena C, editors. Tundras: vegetation, wildlife and climate trends New York: Nova Publishers. pp. 1–46.

[30] Walker DA, Raynolds MK, Daniels FJA, Einarsson E, Elvebakk A, et al (2005) The Circumpolar Arctic vegetation map. Journal of Vegetation Science 16: 267–282.

[31] Zhitkov BM (1912) Birds of Yamal Peninsula. Yearbook of Zoological museum of Academy of Sciences 17: 311–369.

[32] Sdobnikov VM (1937) Distribution of mammals and birds on habitats on Bolshezemelskaya tundra and Yamal. Proceedings of the Allunion Arctic institute 94: 1–76.

[33] Uspenskiy SM (1960) Latitudinal zonality of the Arctic avifauna Ornithologia. 55–70.

[34] Danilov NN, Ryzhanovskiy VN, Ryabitsev VK (1984) Birds of Yamal. Moscow: Nauka. 333 p.

[35] Baril L, Hansen A, Renkin R, Lawrence R (2009) Willow-bird relationships on Yellowstone's northern range. Yellowstone Science 17: 19–26.

[36] Ripple WJ, Beschta RL (2005) Refugia from browsing as reference sites for restoration planning. Western North American Naturalist 65: 269–273.

[37] Shiyatov SG, Mazepa VS (1995) Climate. In: Dobrinskiy LN, editor. The nature of Yamal. Yekaterinburg: Nauka. pp. 32–68.

[38] Magomedova MA, Morozova LM, Ektova SN, Rebristaya OV, Chernyadyeva IV, et al.. (2006) Yamal peninsula: vegetation cover; Gorchakovskiy PL, editor. Tumen: City-press. 360 p.

[39] Chernov Y, Matveyeva N (1997) Arctic ecosystems in Russia. In: Wielgolaski F, editor. Ecosystems of the World. Amsterdam: Elsevier. pp. 361–507.

[40] den Herder M, Virtanen R, Roininen H (2004) Effects of reindeer browsing on tundra willow and its associated insect herbivores. Journal of Applied Ecology 41: 870–879.

[41] Ims RA, Yoccoz NG, Brathen KA, Fauchald P, Tveraa T, et al (2007) Can reindeer overabundance cause a trophic cascade? Ecosystems 10: 607–622.

[42] Freedman B, Svoboda J (1982) Populations of breeding birds at Alexandra Fjord, Ellesmere Island, Northwest Territories, compared with other arctic localities. Canadian Field-Naturalist 96: 56–60.

[43] Tomialojc L, Verner J (1990) Do point counting and spot mapping produce equivalent estimates of bird densities. Auk 107: 447–450.

[44] Trefry SA, Freedman B, Hudson JMG, Henry GHR (2010) Breeding bird surveys at Alexandra Fjord, Ellesmere Island, Nunavut (1980–2008). Arctic 63: 308–314.

[45] Dray S, Chessel D, Thioulouse J (2003) Co-inertia analysis and the linking of ecological data tables. Ecology 84: 3078–3089.

[46] ter Braak CJF (1986) Canonical correspondence-analysis - a new eigenvector technique for multivariate direct gradient analysis. Ecology 67: 1167–1179.

[47] Greenacre M (2010) Correspondence analysis of raw data. Ecology 91: 958–963. 20462111 10.1890/09-0239.1

[48] Chessel D, Lebreton J-D, Yoccoz N (1987) Propriétés de l'Analyse Canonique des Correspondances; une illustration en hydrobiologie. Revue de Statistique Appliquée 35: 55–72.

[49] Julliard R, Clavel J, Devictor V, Jiguet F, Couvet D (2006) Spatial segregation of specialists and generalists in bird communities. Ecology Letters 9: 1237–1244. 17040326 10.1111/j.1461-0248.2006.00977.x

[50] Danilov NN (1966) The ways of adaptation of land vertebrate animals to living conditions in Subarctic. Proceedings of the Institute of Biology of UD RAS 2. Birds 1–147.

[51] Gotelli NJ, Colwell RK (2001) Quantifying biodiversity: procedures and pitfalls in the measurement and comparison of species richness. Ecology Letters 4: 379–391.

[52] R Development Core Team (2010) R: A Language and Environment for Statistical Computing. Vienna, Austria: R Foundation for Statistical Computing.

[53] Chessel D (2004) The ade4 package-I: One-table methods. R News 4: 5–10.

[54] Oksanen J, Blanchet GF, Kindt R, Legendre P, O'Hara RB, et al (2010) Vegan: Community Ecology Package. R package 117-4. 1.17-4 ed.

[55] Jackson CR, Robertson MP (2011) Predicting the potential distribution of an endangered cryptic subterranean mammal from few occurrence records. Journal for Nature Conservation 19: 87–94.

[56] Rodrigues R (1994) Microhabitat variables influencing nest-site selection by tundra birds. Ecological Applications 4: 110–116.

[57] Kucheruk VV, Kovalevskiy YV, Surbanos AG (1975) Changes in bird populations and fauna of southern Yamal during the last 100 years. Bulletin Moscow National Society, Department of Biology 80: 52–64 (in Russian).

[58] Ryabitsev VK (1993) Distribution and dynamics of bird communities in the Sub-Arctic. Ekaterinburg (in Russian). Nauka Publisher. 293 p.

[59] Sokolov VA (2006) Comparative analysis of the nesting bird fauna in south-western Yamal. Izvestya Chelyabinskogonauchnogo centra Ural Okrug. RAN 3: 109–113 (in Russian).

[60] Chapin FS, Sturm M, Serreze MC, McFadden JP, Key JR, et al (2005) Role of land-surface changes in Arctic summer warming. Science 310: 657–660. 16179434 10.1126/science.1117368

[61] Naito AT, Cairns DM (2011) Patterns and processes of global shrub expansion. Progress in Physical Geography 35: 423–442.

[62] Clavero M, Villero D, Brotons L (2011) Climate change or land use dynamics: do we know what climate change indicators indicate? Plos One 6. 10.1371/journal.pone.0018581 PMC3080866 21533025

[63] Killengreen S, Lecomte N, Ehrich D, Schott T, Yoccoz NG, et al (2011) The importance of marine vs. human-induced subsidies in the maintenance of an expanding mesocarnivore in the arctic tundra. Journal of Animal Ecology 80: 1049–1060. 21477201 10.1111/j.1365-2656.2011.01840.x

[64] Killengreen ST, Stromseng E, Yoccoz NG, Ims RA (2012) How ecological neighbourhoods influence the structure of the scavenger guild in low arctic tundra. Diversity and Distributions 18: 563–574.

