# Supplementary material for: Bird Communities of the Arctic Shrub Tundra of Yamal: Habitat Specialists and Generalists
Source: PLoS One. 2012 Dec 11;7(12):e50335. doi: 10.1371/journal.pone.0050335 (PMC3519781; doi:10.1371/journal.pone.0050335)
Supplement: Methods S1 — Details about the survey method. (DOC) [file pone.0050335.s001.doc]

# Supporting Methods S1

**Bird communities of the Arctic shrub tundra of Yamal: habitat specialists and generalists**

**Vasiliy Sokolov^1*^, Dorothée Ehrich^2^,** **Nigel G. Yoccoz^2*^,** **Alexander Sokolov^3^,** **Nicolas Lecomte^2**^**

**1** Institute of Plant & Animal Ecology, Ural Division Russian Academy of Sciences, 202 - 8 Marta street, Ekaterinburg, 620144, Russia

* Corresponding authors. E-mail: [vskolov@inbox.ru](mailto:vskolov@inbox.ru); [nigel.yoccoz@uit.no](mailto:nigel.yoccoz@uit.no); nicolas.lecomte@uit.no

**2** Department of Arctic and Marine Biology, University of Tromsø, N-9037 Tromsø, Norway

**3** Ecological Research Station of the Institute of Plant & Animal Ecology, Ural Division Russian Academy of Sciences, 21 Zelyonaya Gorka, Labytnangi, Yamalo-Nenetski district, 629400, Russia

** Current address: Department of Environment, Government of Nunavut, X0A0L0 Igloolik, Canada

**Details about the survey method**

The main survey method was spot mapping [1,2,3]. Each plot was surveyed by walking back and forth at a slow pace along tracks 100 m apart, recording all alarming or singing birds, at least four times in each breeding season. After each survey the locations where breeding pairs were recorded or inferred were plotted on a map for each species. Maps of subsequent surveys were compared and assembled to result in one map of breeding pairs to represent their distribution in that year. In order to map as precisely as possible, discrepancies between the results of different surveys were solved by specifically checking places where uncertainties were arising. Two examples of such maps are shown in figure S2, illustrating the density of common species breeding in open landscapes (A) and in willow thickets (B). The mapping was further improved by using two additional observation methods. For the most common open landscape breeders (Lapland bunting and red-breasted pipit), nest searches were carried out in places where several birds were alarming together. For species breeding in thickets which can be difficult to register precisely by spot mapping, point counts were carried out additionally.

## Supplemental references

1. Freedman B, Svoboda J (1982) Populations of breeding birds at Alexandra Fjord, Ellesmere Island, Northwest Territories, compared with other arctic localities. Canadian Field-Naturalist 96: 56-60.

2. Tomialojc L, Verner J (1990) Do point counting and spot mapping produce equivalent estimates of bird densities. Auk 107: 447-450.

3. Trefry SA, Freedman B, Hudson JMG, Henry GHR (2010) Breeding Bird Surveys at Alexandra Fiord, Ellesmere Island, Nunavut (1980-2008). Arctic 63: 308-314.

4. Sokolov VA (2006) Comparative analysis of the nesting bird fauna in south‐western Yamal. Izvestya Chelyabinskogonauchnogo centra Ural Okrug. RAN 3: 109‐113 (in Russian).
