# Supplementary figures and images for: Bird Communities of the Arctic Shrub Tundra of Yamal: Habitat Specialists and Generalists
Source: PLoS One. 2012 Dec 11;7(12):e50335. doi: 10.1371/journal.pone.0050335 (PMC3519781; doi:10.1371/journal.pone.0050335)

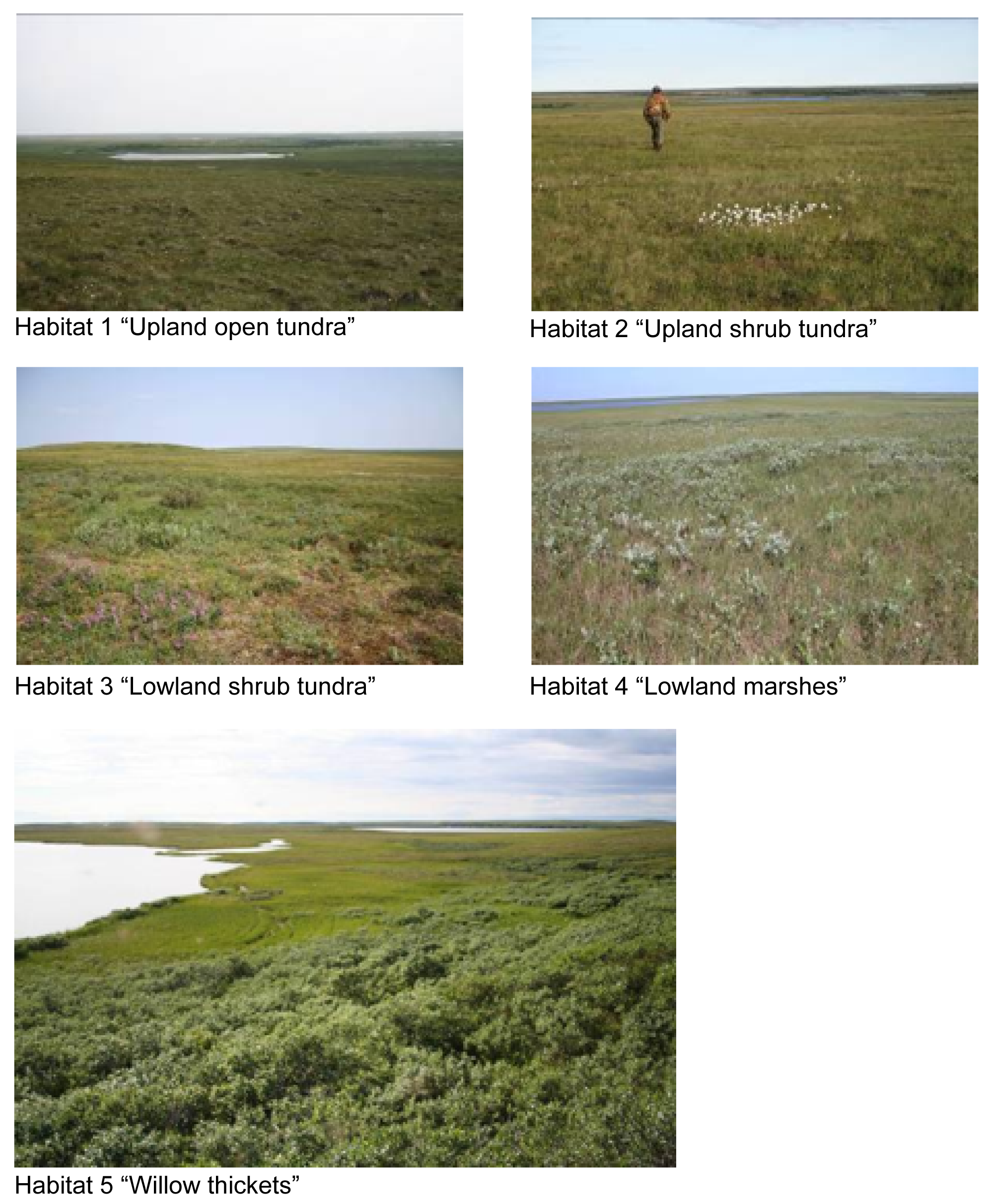

Supplement: Figure S1 — Summer pictures of the habitats monitored in our study, Erkuta, 2002–2009, Yamal, Russia. (TIF) [file pone.0050335.s002.tif]

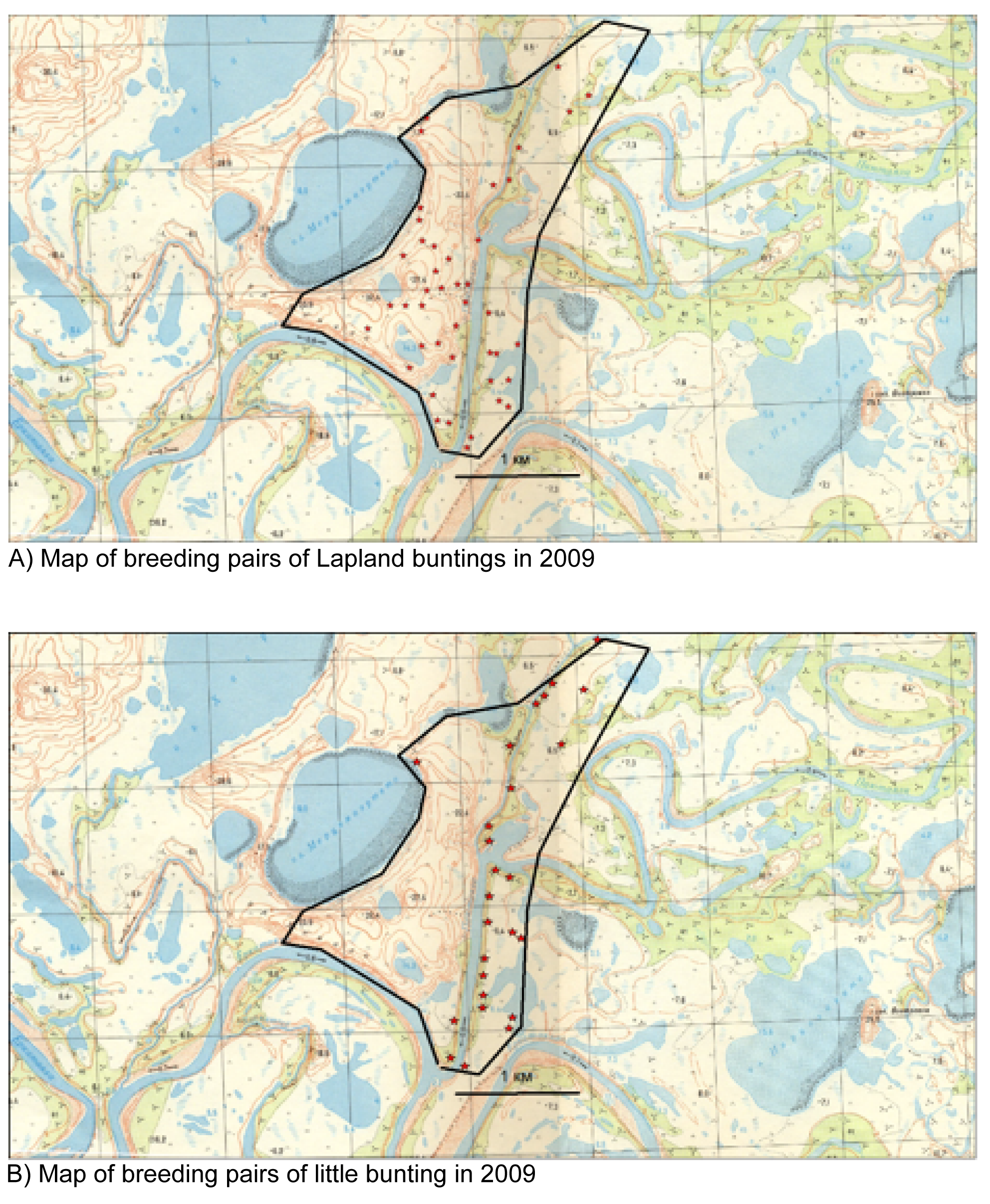

Supplement: Figure S2 — Map of breeding pairs for two species breeding in open habitat (Lapland bunting) and closed habitats (willow thickets; little bunting). Each star represents the centre of a territory. (TIF) [file pone.0050335.s003.tif]

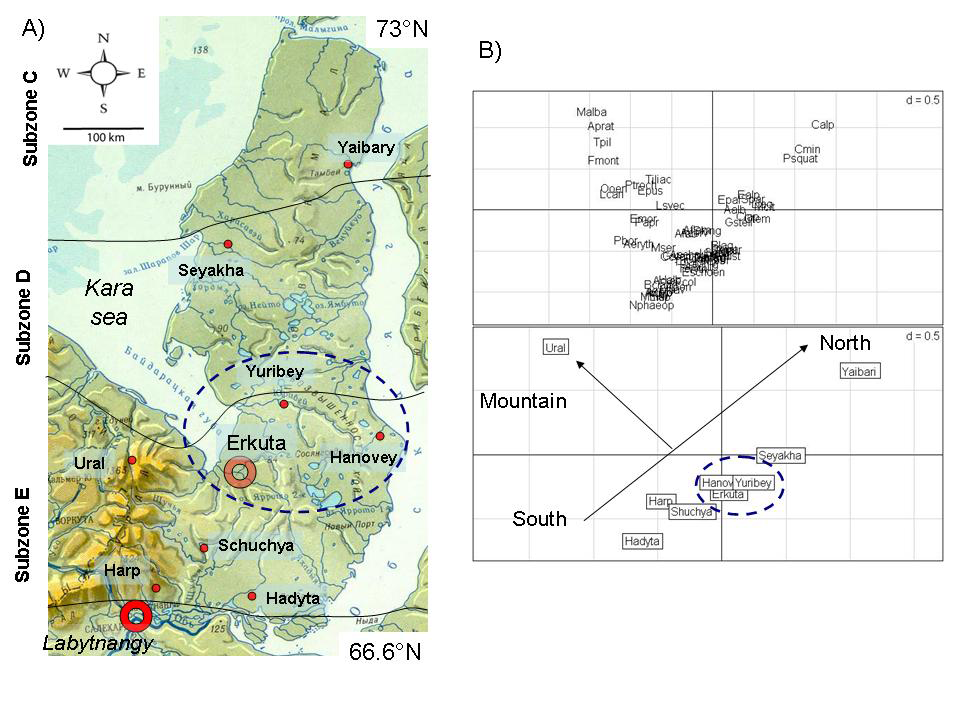

Supplement: Figure S3 — Bird communities have been described at several sites on the Yamal Peninsula by different authors. A) Map of the sites where communities were described. B) Result of a correspondence analysis which shows that the community at Erkuta was similar to those observed at sites located in the same biogeographic area, such as Hanovey and Yuribey (Sokolov et al. 2006). (TIF) [file pone.0050335.s004.tif]
